# Comparison of frontal alpha asymmetry among schizophrenia patients, major depressive disorder patients, and healthy controls

**DOI:** 10.1186/s12888-020-02972-8

**Published:** 2020-12-10

**Authors:** Kuk-In Jang, Chany Lee, Sangmin Lee, Seung Huh, Jeong-Ho Chae

**Affiliations:** 1 Cognitive Science Research Group, Korea Brain Research Institute, Daegu, South Korea; 2 Department of Psychiatry, College of Medicine, The Catholic University of Korea, 222 Banpo-daero, Seocho-gu, Seoul, 137-701 South Korea

**Keywords:** Frontal alpha asymmetry, Electroencephalography, Depression, Schizophrenia

## Abstract

**Background:**

Electroencephalography (EEG) frontal alpha asymmetry (FAA) has been observed in several psychiatric disorders. Dominance in left or right frontal alpha activity remains inconsistent in patients with major depressive disorder (MDD), patients with schizophrenia, and healthy controls. This study compared FAA among patients with MDD and schizophrenia, and healthy controls.

**Methods:**

We recruited 20 patients with MDD, 18 patients with schizophrenia, and 16 healthy individuals. The EEG alpha frequency ranged from 8 Hz to 12 Hz. FAA was expressed as the difference between absolute power values of right and left hemisphere electrodes in the alpha frequency range (common-log-transformed frontal right- and left-hemisphere electrodes: F4–F3, F8–F7, FP2–FP1, AF4–AF3, F6–F5, and F2–F1). Hamilton depression and anxiety rating scales were evaluated in patients with MDD. Positive and negative syndrome scales were evaluated in patients with schizophrenia.

**Results:**

Patients with schizophrenia showed significantly lower left FAA than healthy controls (F4–F3, schizophrenia vs. healthy controls: − 0.10 ± 0.04 vs. -0.05 ± 0.05). There were no significant differences in FAA between patients with schizophrenia and MDD as well as between patients with MDD and healthy controls.

**Conclusions:**

The present study suggests that FAA indicates a relatively lower activation of left frontal electrodes in schizophrenia. The left-lateralized FAA could be a neuropathological attribute in patients with schizophrenia, but a lack of sample size and information such as medication and duration of illness might obscure the interpretation and generalization of our findings. Thus, further studies to verify the findings would be warranted.

**Supplementary Information:**

The online version contains supplementary material available at 10.1186/s12888-020-02972-8.

## Background

Although electroencephalographic frontal alpha asymmetry (FAA) has been suggested to be a clinical biomarker for the abnormalities in major depressive disorder (MDD) [1–9], conflicting results in prior research have contested this view [10, 11]. A previous meta-analysis of 1883 individuals with MDD and 2161 healthy controls found that FAA’s diagnostic value was not significant [11]. However, hemispheric lateralization of brain activity could reflect a potential risk underlying neurophysiological attributes in psychiatric disorders such as MDD [12] and schizophrenia [13]. As a model to understand physiologic state, FAA could help to expand our knowledge of schizophrenia and MDD.

Neurobiological abnormalities in depression have been linked to uncontrollable avoidant behavior [14]. The core feature of depressive symptoms is a mood change that influences coping strategies in response to daily life events [15–18]. The approach-withdrawal hypothesis offers one model of such a coping strategy, categorizing an emotional response to an external event in terms of the subsequent actions [19, 20]. It has been hypothesized the two motivational behaviors in response to stimuli [21, 22]: seeking and avoidance. These two behavior systems reflect the frontal hemispheric activations [23–26]: left frontal activation could be considered as an approach system paired with a positive emotion, while right frontal activation could indicate a withdrawal system involving negative emotion [24, 27]. Compared to resting-state right frontal alpha power measured by electroencephalography (EEG), reduced left frontal alpha power reflects an increase in left frontal activity [28].

The withdrawal system has also explained a character of behavior in schizophrenia, which indicates collateral forms of motivational impairment, such as anhedonia and avolition [29]. Individuals with schizophrenia also exhibit a higher left alpha power than right alpha power [30], and these measurements were significantly different from those recorded in healthy controls [10]. In the above study, subjects with schizophrenia exhibited a tendency toward left lateralized alpha power compared to those with MDD, post-traumatic stress disorder, panic disorder, attention deficit hyperactivity disorder, and conduct disorder, while those with MDD exhibited a tendency toward right lateralized alpha power. However, a meta-analysis study investigating FAA has inconsistent findings [11]. Furthermore, studies comparing subjects with MDD and with other psychiatric disorders, such as schizophrenia, are scarce. In neurophysiologic view of approach and withdrawal system, lateralization of brain activity could explain pathologic state between patients with MDD and schizophrenia. This study sought to explain these inconsistencies in the literature by comparing FAA between MDD patients, schizophrenia patients, and healthy controls.

Although the present study focuses on comparisons of asymmetric alpha band power (8 Hz to 12 Hz) at frontal region between patients with schizophrenia and MDD, the other EEG asymmetric band powers have implicated to attain comprehensive knowledge about the role of hemispheric activity in the brain. Hypofrontality in patients with schizophrenia was evidenced by a greater activity of EEG delta (1 Hz to 4 Hz) rhythm in left-side frontal brain which was related with delusion [31]. Recently, one study reported a less activation in left frontal brain in patients with MDD, that a lower high-beta (20 Hz to 35 Hz) amplitude in left frontal region was associated with language represents [32]. These findings could have implicated that EEG frontal activity represents the functionality of left- or right-side brain in psychiatric disorders.

The present study hypothesized that significant differences in FAA would be found between MDD patients, schizophrenia patients, and healthy controls. In addition, we hypothesized that patients with schizophrenia would show left-lateralized FAA compared to patients with MDD and healthy individuals.

## Methods

### Participants

This study recruited 20 patients with MDD (11 women), 18 patients with schizophrenia (9 women), and 16 healthy controls (8 women). All participants were native Koreans. Inclusion criteria for the participants were as follows: (1) age ranged 19 to 65 years; (2) in case of patients, met the requirements of the *Diagnostic and Statistical Manual of Mental Disorders, 4th Edition* (DSM-4); (3) normal vision or hearing. Participants and patients with (1) vision or hearing problems, (2) drug and/or alcohol abuse, (3) traumatic brain injury, and (3) a lifetime history of neurological disorders were excluded. Furthermore, healthy subjects with a lifetime history of psychiatric disorders were excluded. Patients and healthy individuals were diagnosed based on the Structured Clinical Interview using the MINI International Neuropsychiatric Interview in the DSM-4. The MINI, a clinician-administered structured interview, was designed to measure anxiety, mood, eating, substance use, and psychotic disorders. According to DSM-4 criteria, and patients with MDD and schizophrenia were diagnosed. Clinical symptoms were evaluated by a trained psychiatrist. Hamilton Depression and Anxiety [33, 34] rating scales were evaluated in patients with MDD. Positive and Negative Syndrome Scales [35] were evaluated in patients with schizophrenia. Healthy participants were recruited through public advertising in Seoul, Korea. The mean (± SD) age of all participants was 37.63 ± 11.38 years (range, 19–59 years). The present study was conducted in compliance with the principles of the Declaration of Helsinki and was approved by the Institutional Review Board of Seoul St. Mary’s Hospital, College of Medicine, The Catholic University of Korea (approval number KC14DDSE0479). All participants provided written informed consent. All experimental procedures followed relevant institutional guidelines and regulations.

### Electrophysiological measurement and analysis

Participants were seated in a comfortable chair in a sound-attenuated room. EEG data were recorded using an amplifier (NeuroScan SynAmps Compumedics USA, El Paso, TX, USA) with a headcap equipped with AgCl electrodes according to the international 10–20 system. We used an EEG device that records from 62 scalp positions—15 standard channels (FP1, FP2, F7, F3, FZ, F4, F8, C3, CZ, C4, P3, PZ, P4, O1, and O2) and 47 extended channels (FPZ, AF3, AF4, F5, F1, F2, F6, FT7, FC5, FC3, FC1, FCZ, FC2, FC4, FC6, FT8, T7, C5, C1, C2, C6, T8, TP7, CP5, CP3, CP1, CPZ, CP2, CP4, CP6, TP8, P7, P5, P1, P2, P6, P8, PO7, PO5, PO3, POZ, PO4, PO6, PO8, CB1, OZ, and CB2). Additional electrodes were placed above and below the left eye for vertical electrooculography (VEO) and at the outer canthus of each eye for horizontal electrooculography. EEG data were recorded using a 0.1–100 Hz bandpass filter at a sampling rate of 1000 Hz. The signals were referenced to both mastoids, and the ground electrode was placed on the forehead. The impedance between the electrodes and the scalp was maintained below 5 kΩ during the entire recording session. Subsequently, the EEGs were preprocessed using Scan 4.5 software and Curry 7.0 (Compumedics USA, El Paso, TX, USA). Gross artifacts were rejected through visual inspection of the recording by a trained individual who had no previous information regarding the data origin.

### Resting state EEG paradigm and alpha asymmetry calculation

**Fig. 1:**
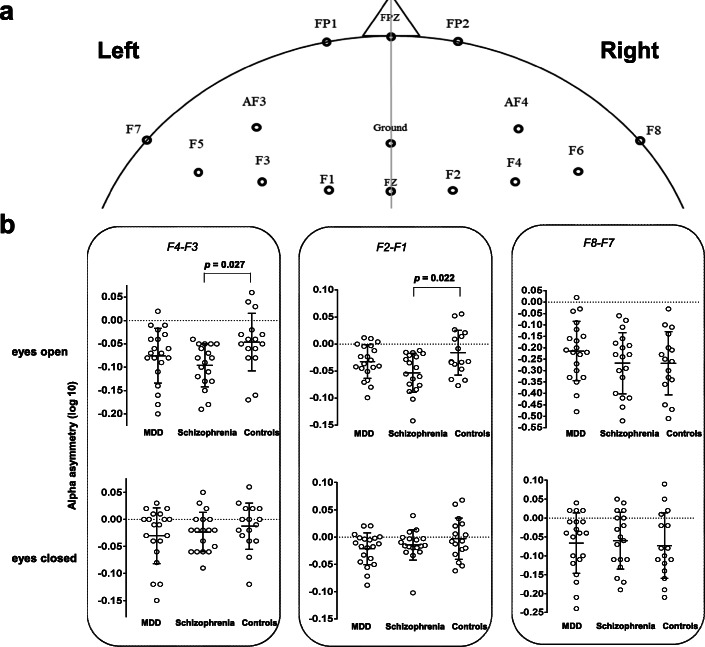
Comparison of alpha asymmetry among MDD patients, schizophrenia patients, and healthy controls. **a** shows frontal electrodes sites. **b** shows comparisons of alpha asymmetries observed across comparisons. Error bars indicate mean ± standard deviation

Resting EEG was recorded with eyes open and closed for 5 min each. Eye blinking artifacts can have an undesirable effect on EEG band power, and therefore they were corrected using established mathematical procedures [36, 37]. Additionally, based on VEO, positive and negative components exceeding 300 μV from before and after a maximum peak of blinking interval (− 100 ms to 300 ms) in the frontal regions were considered covariant. EEGs were analyzed using Matlab 2016 software (Mathworks, Inc., Natick, MA, USA) including a fast Fourier transform with a 1–50 Hz bandpass filter to calculate the absolute power in delta (1 Hz to 4 Hz), theta (4 Hz to 8 Hz), alpha (8 Hz to 12 Hz), beta (12 Hz to 30 Hz), and gamma (30 Hz to 50 Hz) bands . The power values were displayed as averaged points in the frequency range. Artifacts exceeding ±100 μV were rejected at all electrode sites. For each participant, 30 randomized artifact-free epochs (epoch length 2.048 s) were used in the analysis. The F4 and F3 electrodes covered the middle-frontal scalp region, while the F8 and F7 electrodes covered the lateral-frontal scalp areas, which are associated with frontal alpha asymmetry for depressive disorder (Fig. 1a) [11]. Additionally, four electrodes pairs were also included in sub-analysis: FP2-FP1, AF4-AF3, F6-F5, and F2-F1. Delta band frequency was considered as ensuring the effect of residual ocular artifact on the present results (Supplementary Table 1). To normalize the FAA data, a common log transformation was applied to the power values of selected electrodes [38]. FAA has been defined as hemispheric differences [39], which was calculated as the difference between selected electrodes, right frontal alpha power, and left frontal alpha power [40–44]. More negative value of FAA indicates a relatively higher alpha activity in left frontal brain as low metabolic brain activations of left-side. To calculate power spectrum (*PS*), the discrete Fourier transformation analysis was performed [45], where *s* is the time series and *N* is the epoch size which is 2048 in this study. *FAA* can be calculated by $FAA={\mathit{\log}}_{10}{PS}_R^{\alpha }-{\mathit{\log}}_{10}{PS}_L^{\alpha }$,where ${PS}_R^{\alpha }$ and ${PS}_L^{\alpha }$ are alpha power of EEG signals at right and left electrodes, respectively. Hence, FAA can be obtained using the electrode pairs F4-F3, F8-F7, FP2-FP1, AF4-AF3, F6-F5, and F2-F1.

$$ PS\left[m\right]=\sum \limits_{n=0}^{N-1}s\left[n\right]{e}^{-\frac{j2\pi mn}{N}} $$

### Statistical analysis

Demographic statistics with age and sex between participant groups were tested using analysis of variance (ANOVA) or chi-squared tests. Comparisons of alpha asymmetry were performed using multivariate analysis of covariance. Within-subject factors included alpha asymmetry values (log-transformed right-side electrode–left-side electrode at frontal lobe) with eyes open and closed. The groups constituted the between-subject factors. Age and sex were considered as covariates. Partial correlations between alpha asymmetry and clinical symptoms were analyzed to account age and sex. Bootstrapping test was performed in the correlation analysis, and the sampling number was 10,000, which has been accepted in previous studies [46–48]. Alpha asymmetry between men and women was compared using ANOVA. The *p*-values were corrected using the Bonferroni method, which applied to multiple comparisons of several experimental conditions and variables [49, 50].

## Results

The mean age ranges in the groups were 42.60 ± 11.48 in patients with MDD, 32.00 ± 10.45 in patients with schizophrenia, and 37.75 ± 9.78 in healthy controls. Descriptive characteristics of study participants were summarized in Table 1. The group difference in age was significant between patients with MDD and those with schizophrenia (*f* = 4.68, *p* = 0.014). Individual demographic data and clinical symptom scores of each participant were presented in Table 2. In delta band frequency with eyes-opened condition, none of residual ocular artifact was confirmed through no significant differences of delta power between participant groups (Supplementary Table 1).

**Table 1:** Descriptive characteristics of the study group

Variable	MDD (a) (*n* = 20)	Schizophrenia (b) (*n* = 18)	Control (c) (*n* = 16)	Statistics
Age (years)	42.60 ± 11.48	32.00 ± 10.45	37.75 ± 9.78	*f* = 4.68; *p* = 0.014; a > b
Sex (male/female), n/n	9/11	9/9	8/8	*χ*^2^ = 0.13, *p* = 0.939
HAM-D	23.70 ± 4.86	–	–	–
HAM-A	20.45 ± 7.39	–	–	–
PANSS-Positive	–	30.33 ± 5.34	–	–
PANSS-Negative	–	17.67 ± 6.05	–	–
PANSS-General	–	52.78 ± 8.62	–	–
PANSS-Total	–	100.78 ± 11.70	–	–

Data presented as mean ± SD unless otherwise indicated

*MDD* Major depressive disorder, *HAM-D* Hamilton-Depression scale, *HAM-A* Hamilton Anxiety scale, *PANSS* Positive and Negative Syndrome Scale

**Table 2:** Demographics and clinical scores in the individual participants

MDD					Schizophrenia							Controls		
Individuals	Age range	Sex	Depression	Anxiety	Individuals	Age range	Sex	Positive	Negative	General	Total	Individuals	Age range	Sex
D01	40–45	m	26	34	S01	19–25	m	32	16	63	111	C01	30–35	m
D02	30–35	f	22	23	S02	35–40	f	31	28	40	99	C02	40–45	f
D03	30–35	f	22	25	S03	25–30	f	32	18	48	98	C03	45–50	f
D04	19–25	f	9	10	S04	50–55	f	31	20	53	104	C04	40–45	m
D05	45–50	f	32	35	S05	30–35	m	38	14	53	105	C05	35–40	f
D06	35–40	m	29	35	S06	19–25	f	23	17	58	98	C06	30–35	f
D07	40–45	f	21	22	S07	25–30	f	29	12	48	89	C07	35–40	f
D08	19–25	m	29	23	S08	19–25	m	31	17	50	98	C08	25–30	m
D09	40–45	m	24	22	S09	19–25	m	21	16	41	78	C09	50–55	m
D10	55–60	f	27	23	S10	30–35	m	34	11	46	91	C10	25–30	m
D11	40–45	f	19	14	S11	35–40	f	36	10	59	105	C11	19–25	f
D12	55–60	m	21	13	S12	30–35	f	33	15	64	112	C12	19–25	m
D13	55–60	m	21	11	S13	19–25	m	35	17	67	119	C13	45–50	m
D14	50–55	m	22	17	S14	25–30	m	19	35	66	120	C14	50–55	m
D15	35–40	f	25	18	S15	55–60	f	31	19	43	93	C15	40–45	f
D16	30–35	f	26	15	S16	19–25	m	24	16	49	89	C16	45–50	f
D17	40–45	m	21	18	S17	45–50	m	36	23	57	116			
D18	55–60	f	24	16	S18	35–40	f	30	14	45	89			
D19	40–45	m	28	17										
D20	55–60	f	26	18										

*MDD* Major depressive disorder

An interaction effect, accounting for age and sex, was not significant between group and FAA (*f* = 1.30, *p* = 0.253, η_p_^2^ = 0.100). However, for between-subjects effects (schizophrenia vs. healthy control), we observed a significant difference in F4–F3 with the eyes-opened condition (*f*_[2, 49]_ = 3.70, *p* = 0.032, η_p_^2^ = 0.131). Alpha asymmetry in the schizophrenia group was lower than that in the healthy controls (− 0.10 ± 0.04 vs. -0.05 ± 0.05, corrected *p* = 0.027, 95% CI = 0.01 to 0.10) (Fig. 1b and Table 3). There were no significant differences in F4–F3 with the eyes-opened condition between patients with MDD and healthy controls (corrected *p* = 0.630, 95% CI = − 0.02 to 0.07), or between MDD and schizophrenia patients (corrected *p* = 0.434, 95% CI = − 0.02 to 0.08). Furthermore, there were no significant differences in F4–F3 with eyes-closed (*f*_[2, 49]_ = 0.64, *p* = 0.532, η_p_^2^ = 0.025), or in F8–F7 with eyes-opened or -closed (*f*_[2, 49]_ = 0.96, *p* = 0.391, η_p_^2^ = 0.038; *f*_[2, 49]_ = 0.11, *p* = 0.896, η_p_^2^ = 0.004) among participant groups. In sub-analysis, between patients with schizophrenia and healthy controls, a significant difference of FAA was found in F2–F1 with eyes-opened (*f*_[2, 49]_ = 3.93, *p* = 0.026, η_p_^2^ = 0.138) (Table 3). Patients with schizophrenia showed a lower FAA in comparison with healthy controls (− 0.05 ± 0.04 vs. -0.02 ± 0.04, corrected *p* = 0.022, 95% CI = 0.004 to 0.07). There were no significant differences in FP2–FP1, AF4–AF3, and F6–F5 with eyes-opened or -closed, and F2–F1 with eyes-closed (Table 3).

**Table 3:** Comparison of FAA between participant groups

FAA	MDD (a)	Schizophrenia (b)	Control (c)	Statistics	
Eyes-open					
F4-F3	−0.08 ± 0.05	**−0.10 ± 0.04**	**−0.05 ± 0.05**	***p*** **= 0.032**	***p =*** **0.027*, b < c***
F8-F7	−0.22 ± 0.12	−0.27 ± 0.12	−0.27 ± 0.13	*p* = 0.391	
FP2-FP1	−0.11 ± 0.07	−0.14 ± 0.08	−0.14 ± 0.12	*p* = 0.527	
AF4-AF3	−0.08 ± 0.05	− 0.10 ± 0.05	− 0.06 ± 0.08	*p* = 0.185	
F6-F5	−0.16 ± 0.13	−0.19 ± 0.09	−0.15 ± 0.08	*p* = 0.195	
F2-F1	−0.03 ± 0.03	**−0.05 ± 0.04**	**−0.02 ± 0.04**	***p*** **= 0.026**	***p =*** **0.022*, b < c***
Eyes-closed					
F4-F3	−0.03 ± 0.04	−0.02 ± 0.03	−0.01 ± 0.03	*p* = 0.532	
F8-F7	−0.07 ± 0.07	−0.06 ± 0.07	−0.07 ± 0.08	*p* = 0.896	
FP2-FP1	−0.02 ± 0.05	0.003 ± 0.02	−0.01 ± 0.06	*p* = 0.358	
AF4-AF3	−0.03 ± 0.05	−0.03 ± 0.05	−0.02 ± 0.03	*p* = 0.698	
F6-F5	−0.09 ± 0.12	−0.04 ± 0.06	−0.05 ± 0.06	*p* = 0.307	
F2-F1	−0.02 ± 0.03	−0.01 ± 0.03	−0.003 ± 0.04	*p* = 0.238	

Data presented as mean ± SD unless otherwise indicated

*FAA* Frontal alpha asymmetry, *MDD* Major depressive disorder

In correlation analysis, there were no significant associations between alpha asymmetry and clinical symptoms (depression and F4–F3 eyes-opened, *r* = − 0.29, *p* = 0.246; anxiety and F4–F3 eyes-opened, *r* = − 0.22, *p* = 0.375; depression and F2–F1 eyes-opened, *r* = 0.05, *p* = 0.839; anxiety and F2–F1 eyes-opened, *r* = − 0.08, *p* = 0.750; schizophrenia-positive and F4–F3 eyes-opened, *r* = − 0.16, *p* = 0.567; schizophrenia-negative and F4–F3 eyes-opened, *r* = − 0.05, *p* = 0.852; schizophrenia-general and F4–F3 eyes-opened, *r* = − 0.26, *p* = 0.330; schizophrenia-total and F4–F3 eyes-opened, *r* = − 0.29, *p* = 0.277; schizophrenia-positive and F2–F1 eyes-opened, *r* = − 0.37, *p* = 0.162; schizophrenia-negative and F2–F1 eyes-opened, *r* = − 0.01, *p* = 0.983; schizophrenia-general and F2–F1 eyes-opened, *r* = − 0.15, *p* = 0.587; schizophrenia-total and F2–F1 eyes-opened, *r* = − 0.27, *p* = 0.308).

## Discussion

The present study quantitatively compared electroencephalographic FAA among MDD patients, schizophrenia patients, and healthy controls. Our results indicated that patients with schizophrenia exhibited a lower alpha asymmetry than healthy participants, and this difference was significant when alpha asymmetry recording was conducted under eyes-opened conditions. Our findings concerning FAA in patients with schizophrenia are supported by a previous study, which showed that patients with schizophrenia had reduced alpha asymmetry of functional connectivity than healthy controls [51]. A lower alpha activity which a low brain activation at left frontal region could be implicated that malfunctions in the positive emotional or behavioral approach system of left frontal brain are dominant in patients with schizophrenia. On the other hand, deeply carved approaches with negative emotion or behavior corresponding to right frontal activation could be a representative pathological attribute of schizophrenia.

Although the design of the present study focused on the identification of between-subjects effects, none of significant results found between MDD patients and healthy controls, or between MDD patients and schizophrenia patients. This lack of significance might be attributed to the small sample size, which make it difficult to generalize the results. Potential limitations of our small sample size was revealed by statistical analysis and data processing as well as a lack of information such as duration of illness and pharmacological history. Thus, these should be taken into consideration when interpreting the present findings. There were no associations between FAA and clinical symptoms, and it should be also interpreted carefully. Withdrawal motivation in patients with depression and schizophrenia is closely related to relative increases in right frontal brain activation or relative decreases in right alpha activity [30, 52]. However, the present study showed an absence of measurement in withdrawal and avoidance behavior that should be taken into consideration. The balance of interhemispheric activity may play a role in maintaining mental health across the neurodevelopment of schizophrenia [53], and our study findings partially support this hypothesis. Schizophrenic patients have been shown to exhibit more breaking of rhythmic activity as part of left alpha dominance, compared to healthy participants. Previous studies have also reported that patients with schizophrenia exhibit hyper-activation at high-frequency alpha network in the left frontal area during working memory tasks [54]. Additionally, the present study concerning alpha asymmetry has implications for high stability representing alpha asymmetry recorded when the patient’s eyes were open that was a more useful predictor of disease specificity than data gathered under eyes-closed conditions [55], although eyes-opened condition includes blinking noise which should be carefully handled in preprocessing with artifact removal. Furthermore, the present study showed a significant effect during eyes-opened conditions. In the present finding, variation of FAA scores with eyes-opened was larger than those with eyes-closed as well as eyes-closed alpha activity leading to reduced baseline levels of brain activity compared to eyes-opened activity [56]. Alpha asymmetry in the mid-frontal area is commonly observed in patients with psychiatric disorders [21, 30]. Metabolic and structural alterations in the mid-frontal region are thought to be dominant in patients with schizophrenia [57]. In addition, low-alpha band asymmetry (8 Hz to 10 Hz) was associated with cognitive deficits in patients with MDD and positively correlated with suicidal behavior in the left-side dominant group [58]. Exploring the effect of asymmetric alpha sub-band power on several psychiatric disorders would be helpful to understand brain hemispheric activity completely. Future studies should conduct the association between cognitive deficits and alpha sub-band power asymmetry in patients with schizophrenia.

None of differences were found between patients with MDD and healthy individuals. It has been suggested that FAA does not work as a biomarker to differentiate patients with MDD and patients with non-MDD or healthy controls [59]. Some of findings showed that FAA could be more specific for treatment response of medication [59, 60]. Furthermore, FAA was involved in the risky trait such as a suicidal behavior or ideation in patients with MDD [58, 61]. These studies hereby concluded that FAA might be a prognostic biomarker to assess neurophysiological progressions in patients with MDD, but not to differentiate patients with MDD and healthy individuals.

The present study had several limitations: we lacked patient information regarding medication, the age at onset of the disorder, handedness, behavioral assessment, and level of (formal) education. All these factors could have affected FAA and, therefore, could have influenced our results. In addition, our sample size was insufficient to generalize our findings. This study had an absence of consistent assessment in clinical symptoms and withdrawal/avoidance behavior. Future studies should use clinical scales that consistently evaluate withdrawal/avoidance in all participant groups. Although we suspect that a cross-sectional study may replicate some of our clinical findings, a longitudinal study that investigates alpha asymmetry in a larger cohort would help to verify and expand upon our findings.

## Conclusion

FAA may be a useful neurophysiological biomarker to distinguish between patients with schizophrenia and healthy individuals. Although our findings may have been underpowered due to the small sample size, our present findings suggest that neurobiological abnormalities in FAA are remarkably presented by a left lateralized alpha activity of the patients with schizophrenia.

## Supplementary Information

**Additional file 1: Supplementary Table 1.** Comparison of frontal delta power between participant groups.

## Data Availability

Data supporting our findings are available from the corresponding author on reasonable request.

## Abbreviations

ANOVA: Analysis of variance
EEG: Electroencephalography
FAA: Frontal alpha asymmetry
MDD: Major depressive disorder
VEO: Vertical electro-oculography
PS: Power spectrum
