# Supplementary material for: Comparison of frontal alpha asymmetry among schizophrenia patients, major depressive disorder patients, and healthy controls
Source: BMC Psychiatry. 2020 Dec 10;20:586. doi: 10.1186/s12888-020-02972-8 (PMC7727195; doi:10.1186/s12888-020-02972-8)
Supplement: Supplementary file 1 — Additional file 1: Supplementary Table 1. Comparison of frontal delta power between participant groups. [file 12888_2020_2972_MOESM1_ESM.docx]

**Supplementary Table 1.** **Comparison of frontal delta power between participant groups**

| FAA | MDD (a) | | Schizophrenia (b) | Control (c) | Statistics | |
| --- | --- | --- | --- | --- | --- | --- |
| Eyes-open | |  |  |  |  |  |
| F4-F3 | | -0.10 ± 0.09 | -0.08 ± 0.15 | -0.09 ± 0.10 | *p*=0.954 |  |
| F8-F7 | | -0.28 ± 0.19 | -0.27 ± 0.23 | -0.38 ± 0.32 | *p*=0.394 |  |
| FP2-FP1 | | -0.10 ± 0.11 | -0.15 ± 0.19 | -0.11 ± 0.18 | *p*=0.555 |  |
| AF4-AF3 | | -0.11 ± 0.11 | -0.14 ± 0.19 | -0.10 ± 0.28 | *p*=0.618 |  |
| F6-F5 | | -0.23 ± 0.16 | -0.22 ± 0.19 | -0.16 ± 0.22 | *p*=0.443 |  |
| F2-F1 | | -0.05 ± 0.06 | -0.05 ± 0.07 | -0.04 ± 0.09 | *p*=0.924 |  |
| Eyes-closed | |  |  |  |  |  |
| F4-F3 | | -0.04 ± 0.11 | 0.002 ± 0.14 | -0.02 ± 0.09 | *p*=0.492 |  |
| F8-F7 | | -0.17 ± 0.22 | -0.08 ± 0.12 | -0.12 ± 0.20 | *p*=0.413 |  |
| FP2-FP1 | | -0.04 ± 0.10 | 0.0003 ± 0.06 | -0.01 ± 0.22 | *p*=0.782 |  |
| AF4-AF3 | | -0.06 ± 0.12 | -0.08 ± 0.14 | 0.02 ± 0.20 | *p*=0.085 |  |
| F6-F5 | | **-0.10 ± 0.16** | -0.06 ± 0.10 | **0.04 ± 0.18** | ***p*=0.026** | ***p*=0.023*, a<c*** |
| F2-F1 | | -0.01 ± 0.07 | -0.01 ± 0.06 | -0.01 ± 0.05 | *p*=0.934 |  |

Data presented as mean ± SD unless otherwise indicated.
MDD, Major depressive disorder.
